# The Forgotten (Invisible) Healthcare Heroes: Experiences of Canadian Medical Laboratory Employees Working During the Pandemic

**DOI:** 10.3389/fpsyt.2022.854507

**Published:** 2022-03-16

**Authors:** Basem Gohar, Behdin Nowrouzi-Kia

**Affiliations:** ^1^Department of Population Medicine, The University of Guelph, Guelph, ON, Canada; ^2^Centre for Research in Occupational Safety & Health, Laurentian University, Sudbury, ON, Canada; ^3^Department of Occupational Therapy and Occupational Science, The University of Toronto, Toronto, ON, Canada

**Keywords:** medical laboratory professionals, COVID-19, mental health, Ontario, Canada, qualitative

## Abstract

**Objective:**

The purpose of this qualitative study was to understand the stressors and coping strategies of medical laboratory technologists (MLTs) and assistants (MLAs) working during the COVID-19 pandemic in Ontario, Canada.

**Methods:**

In this descriptive qualitative study, we held two focus groups with MLTs and MLA who were working during the COVID-19 pandemic. The focus group sessions were transcribed verbatim followed by thematic analysis to develop codes and themes.

**Findings:**

A total of 13 participants across Ontario were involved in our study, which included one MLT (*n* = 7) group and one MLA (*n* = 6) MLA. Overall, the stressors and coping methods identified between both focus groups were consistent. Our results revealed four main themes: (1) COVID-19 contributing to the notable and existing staff shortage; (2) the pandemic reinforced that medical laboratory employees are forgotten within the healthcare system; (3) a poor work environment exacerbated by the pandemic; and (4) a resilient and passionate group. Rich descriptions explained the underlying issues related to the themes.

**Conclusions:**

MLTs and MLAs are critical members of the healthcare team and provide vital patient care services. This study explored their experiences working during the pandemic and offers timely recommendations to mitigate against occupational stressors.

## Introduction

The coronavirus disease (COVID-19) has undoubtedly affected healthcare workers' mental health and wellbeing (1). Despite the remarkable effort in containing the virus through physical distancing, restrictions, and vaccinations, various strands of the virus continue mutating, increasing the number of COVID-19 cases and hospital admissions. As a result, the impact of the virus continues to impact frontline healthcare staff like physicians and nurses who have been working longer and harder. Unsurprisingly, the added demand has been impacting their psychosocial wellbeing. Consequently, there have been reports of higher burnout rates, increased sickness absenteeism, and intention to leave the profession (2–4).

We thank our doctors and nurses for their dedication and care as we combat this virus with immense sincerity. Nevertheless, notably, one group of healthcare workers appears to have been forgotten during this pandemic: our medical laboratory technologists (MLTs) and assistants (MLTAs). These workers provide vital services in disease prevention (5). MLTs and MLAs collect and analyze various specimens for diagnostic and treatment purposes. In addition, they play an integral role in COVID-19 virus testing and analysis. In 2021, Ontario medical laboratory personnel managed to collect and analyze 13,262,409 COVID-19 tests, reaching as high as 76,000 tests in a single day (6). Naturally, the demand for medical laboratory services has significantly increased due to the pandemic (7).

Our literature review identified some studies addressing the physical safety aspect of the profession (8, 9). However, despite their essential role in the healthcare system, very little is known about their stressors and experiences during the pandemic. The limited research about their stressors pre-pandemic is equally concerning, suggesting that compared to other healthcare workers, MLTs, and MLAs are vastly understudied. From a provincial perspective, data suggests a shortage in medical laboratory employees before the pandemic (7), which could adversely affect their wellbeing. Recognizing their essential role in healthcare, especially during the pandemic, it is critical to learn about their current stressors and identify stress management strategies. This is of utmost importance since the limited research among MLTs and MLAs point to adverse outcomes such as errors, sickness absenteeism, job dissatisfaction, and intention to leave the profession when work demands are increased (5, 10, 11). These outcomes could have adverse and costly consequences to the general public, as every healthcare system relies heavily on accurate and speedy laboratory results for diagnostics and treatment. Accordingly, the purpose of this qualitative study was to understand the stressors of MLTs and MLAs working during the COVID-19 pandemic in Ontario, Canada.

## Methods

In this descriptive qualitative study, we held focus groups with MLTs and MLAs from Ontario, Canada. We obtained ethics approval from the University of Toronto's Research Ethics Board (REB#00039635). In partnership with the Medical Laboratory Professionals' Association of Ontario, they assisted us with the recruitment of this study. Specifically, they advertised our study through their newsletters and electronic correspondence. Interested participants contacted the authors directly, to which we obtained informed consent in writing and virtually prior to the focus group. Eligible participants were either MLTs or MLAs in Ontario working during the pandemic. MLTs are regulated healthcare providers in Ontario, governed by the College of Medical Laboratory Technologists of Ontario. MLAs include professionals known as phlebotomists, technicians, or assistants. They often assist MLTs in specimen collection for analysis.

Based on the number of interested participants, we held one focus group with MLTs and another with MLAs. It should be noted that MLTs and MLAs have seen a significant increase in work demand due to the pandemic, which could explain the challenges in recruiting participants to form additional focus groups. However, Guest et al. (12) argued that focus groups, as small as two, could reach saturation, capturing up to 80% of themes, including the most prevalent themes.

For health and feasibility reasons, we facilitated the focus groups virtually in June 2021, securely using Microsoft Teams, which were audio-recorded. Two researchers (BG and BNK) were present in each focus group. One author (BG) served as the moderator in both focus groups. Each focus group was ~75 min long. Following the participant introductions, which included information on the participant's region and work setting, we used a semi-structured interviewing process, asking open-ended questions with follow-ups as deemed appropriate (Appendix A). Given the scarcity of information from the literature, we asked about the general stressors in the job then followed up with questions related to pandemic-related stressors. Finally, we asked them how they reduced stress while working during the pandemic. Each participant was assigned a code to ensure confidentiality and accuracy during transcription. The audio recording was later transcribed verbatim into text. The researcher (BG) reviewed the transcripts while listening to the audio recording to improve credibility. This method was also helpful for the data analysis, as it allowed the researcher to be further familiarized with the content (13).

We applied Braun and Clarke's six steps of thematic analysis to develop codes and themes (13). We used Quirkos qualitative data analysis software to code our data (14). Both researchers (BG and BNK) inductively coded the data. Specifically, each researcher read each transcript and coded textual data based on important notes related to the research questions, including similarities and distinctions from each group (13). Based on these patterns, initial codes were produced, and the transcript was reread through, ensuring coding accuracy. Horizontal and vertical relationships were analyzed in forming and clustering codes by which related content was joined to form themes. The codes and themes developed by each researcher were then iteratively reviewed and consolidated accordingly to produce the final themes and subthemes. Both researchers met after coding the data to discuss the codes and themes. The researchers worked on finalizing the themes and subthemes and discussed any discrepancies until a decision had been reached. Additionally, we applied the Consolidated Criteria for Reporting Qualitative Research (COREQ) to ensure the quality of our work (15) (Appendix B).

## Findings

A total of 13 participants were involved in our study. The MLT focus group had seven participants. These included five who identified as women and two who identified as men. The MLA focus group contained six participants who identified as women. Participants were from across Ontario, including northeastern, eastern, southwestern, and central Ontario, including rural and urban regions. There was also diversity in the occupational setting, which included hospitals, private laboratories, and medical health clinics. Please see Table 1 for demographic information.

**Table 1:** Characteristics of participants involved in focus groups.

**Focus group**	**Gender**	**Region**	**Setting**
Medical laboratory technologists Participant coding: G1T#	F = 5; M = 2	Peterborough County = 1 Hamilton Region = 2 Cochrane County = 1 Ottawa Region = 1 Simcoe County = 1 Thames/Middlesex = 1 Identified as working in rural communities = 4	Hospital = 5 Private laboratory = 2
Medical laboratory technicians/assistants Participant coding: G2A#	F = 6	Durham Region = 1 Greater Toronto Area = 3 Niagara Region = 1 Simcoe County = 1 Identified as working in rural communities = 1	Hospital = 3 Private laboratory/doctor's office = 3 Note: some worked multiple jobs in different settings

Overall, both focus groups' stressors and coping methods were reasonably consistent. We identified four primary themes based on our questions and the discussions held in the focus groups, which include (1) COVID-19 contributing to the notable and existing staff shortage; (2) the pandemic reinforced that medical laboratory employees are forgotten within the healthcare system; (3) a poor work environment, exacerbated by the pandemic; and (4) a resilient and passionate group (Table 2). These are discussed in detail in subsequent sections. Each theme contains a table delineating subthemes and selected excerpts.

**Table 2:** Themes and subthemes emerged from focus groups via thematic analysis.

**Themes**	**Subthemes**
Theme 1: COVID-19 contributing to the notable and existing staff shortage	Subtheme 1: Employees are retiring because of the pandemic Subtheme 2: Challenges joining the profession due to limited resources Subtheme 3: Completing tasks that are not within their job role Subtheme 4: History of presenteeism and increased morally injurious situations due to the pandemic Subtheme 5: Absences due to COVID-19
Theme 2: The pandemic reinforced how medical laboratory employees are forgotten within the healthcare system	Subtheme 1: Lack of recognition, gratitude, or compensation compared to other healthcare workers Subtheme 2: Lack of understanding of their roles, contributing to added demands by leaders
Theme 3: Poor work environment exacerbated by the pandemic	Subtheme 1: Unresolved emotions, worsened by the pandemic Subtheme 2: Increased work demand by taking on COVID-19 testing Subtheme 3: Poor communication among healthcare providers and COVID-specific platforms Subtheme 4: Fear of the unknown
Theme 4: A resilient and passionate group	Subtheme 1: Self-care activities during COVID-19, connecting with others, but appreciating alone time Subtheme 2: Means to gaining control despite the pandemic Subtheme 3: Pride and passion for the job

### Theme 1: COVID-19 Contributing to the Notable and Existing Staff Shortage

Staff shortage was identified as a significant contributor to the stress of MLTs and MLAs. Participants reported that staff shortage had been an issue before the pandemic due to high turnover rates (Table 3). However, the negative impact of staff shortage became more profound due to the pandemic. Reportedly, a large proportion of the medical laboratory population is eligible for retirement. As a result, more employees are retiring sooner because of the increased work demand resulting from the pandemic, contributing further to the staff shortage. Additionally, there are few programs and seats within those programs available in Ontario that provide medical laboratory training. Furthermore, there are no “bridging” programs for MLAs who wish to become MLTs. Finally, the ability to complete clinical placements has been more challenging because of the pandemic.

**Table 3:** Theme 1: COVID-19 Contributing to the notable and existing staff shortage.

Subtheme 1: Employees are retiring because of the pandemic	- G2A6 (F): We have people that have been working for the company for over 30 years who have just said like, “forget it. I'm not doing this anymore. This is not the same job as I had before.”
Subtheme 2: Challenges joining the profession due to limited resources	- G1T3 (F): There's no bridging program to move [MLAs] from one world to another without going back to school and starting all over again at square one. Whereas, umm registered practical nurses, there is bridging programs that move them into the RN position. - G2A5 (F): And if there's so much shortage in medical lab technologists, why not push through some of the MLAs and have some staging? - G1T1 (F): A part of it is that there's not enough, um seats in the schools years ago in the 90s. I believe they ended up closing a few labs schools and now, about 45–55% of the workforce is eligible to retire in the next few years and we're not pumping out enough students to fill those gaps that are coming… And again, the schools don't have enough budget to increase the seats. So, it's a big cycle. - G1T4 (F): Especially for COVID, I mean, how many hospitals being short staffed are going to actually accept students right now, right?
Subtheme 3: Completing tasks that not within job description	- G1T6 (F): So now we have to do clerical tasks on top of everything else. - G1T3: There's a shortage of lab technologist and laboratory staff. So oftentimes at these rural areas, we're either working with one other person and sometimes, we're working alone and that can be challenging and poses its own set of safety risks and things like that. So, it's common for us to juggle multiple different rules on any given shift. - G2A6: We are in a small location, so we do about 150 patients a day. I assist the nurse. I have to relieve our greeter when she goes on break.
Subtheme 4: History of presenteeism and increased morally injurious situations due to the pandemic	- G1T1 (F): Before COVID, you would pretty much be expected to come to work unless you were dying and couldn't get out of bed. If you are sick and you call in and sick too many times, but you're legitimately, sick you get put on a sick list and your attendance gets watched and you're almost punished for calling in sick, so people get scared to call in sick because they don't want to end up on the attendance list. But you come to work sick. - G1T1 (F): I have to keep [doing various roles] and help out where I can because if I don't help out, then the patient care suffers and we're here for patients and I don't want patients to suffer so I'll put them before my own needs. So, it's all ‘push yourself to the max', who cares about your mental health? Who cares about your family life? You need to do this for other people and put yourself aside. - G2A3 (F): You can't call in sick. You know, you can't say that you can take your vacation that you booked 6 months ago because we don't have anyone to cover. You can't call in and take a mental health day.
Subtheme 5: Absences due to COVID-19	- We lost staff because of childcare purposes so, coworkers who were parents and again, like this is no fault of their own, they just had to be able to stay home with their kids. - G1T4: I mean, if you've got a staff member in contact with the case, the staff member is now off for 14 days. It's, it's 14 days right now, just in my area. But at my site, we've got a couple off so. And there's nothing we can do but work through it with the staffing we've got. - G2A6: There's nobody to call in so that's the thing so, either everyone is going to absorb that work or someone has to stay late, or someone has come in on a day off and I think you know all of us have worked every day during this whole pandemic.

Given the general shortage in healthcare, particularly in MLTs and MLAs, participants noted that they have been completing several tasks that are not a part of their job description. Some of these tasks include clerical and screening tasks that absorb a significant amount of time from their assigned duties. Juggling several tasks was particularly an area of concern in less populated regions, including rural areas.

Leaving the profession due to issues around childcare because of the pandemic was seen as a stressor contributing to the staff shortage. Additionally, our findings revealed that long absence rates increased because of contracting the virus, causing more staffing disruptions. It is important to note that some participants explained that presenteeism was a significant concern before the pandemic due to the guilt of leaving the team short-staffed. Presenteeism also occurred before the pandemic due to expectations from management, partly due to staff shortage.

Staff shortage was also linked to experiencing poorer mental health, particularly experiencing morally injurious situations. Specifically, MLTs and MLAs find it challenging to take time off due to the increased demands and limited resources. Instead, they feel obligated to work in these fast-paced conditions not to compromise patient care.

### Theme 2: The Pandemic Reinforced That Medical Laboratory Employees Are Forgotten Within the Healthcare System

Our findings suggest that the medical laboratory environment's “behind-the-scenes” nature has led to unique stressors (Table 4). Expressly, participants indicated that they are often forgotten in relation to other healthcare providers. As an example, participants reported that, despite the added demands due to the pandemic, they did not receive expressions of gratitude from the healthcare sector, the public, or the government. Participants also felt forgotten from a financial perspective, where they did not receive “COVID” pay as other essential workers in Ontario. Added stress was incurred when the government indirectly condemned MLTs and MLAs through the media for not producing enough COVID tests. Some participants noted that this “attack” was a clear indication of the lack of understanding from the government about the work of the MLT and MLA population and the increased demands from the pandemic.

**Table 4:** Theme 2: The pandemic reinforced that medical laboratory employees are forgotten within the healthcare system.

Subtheme 1: Lack of recognition, gratitude, or compensation compared to other healthcare workers	- G1T2 (M): There's not really much compensation or any expression of gratitude. - G1T1 (F): We're generally forgotten with everything. For instance, it's lab week this week … But there's been no notification of it from anyone in the hospital other than within the lab. Whereas, nursing week, there's a whole build up to it and you hear about it. It's everywhere. There's newsletters. There's emails. There's prizes. There's tons of stuff. Lab week? Nothing! Another stressor with COVID is the media. So, in the beginning, all the labs had to process this many tests and you have the government saying we're trying to get them to process this many tests, but they're not processing them fast enough. So, when you have people who have no clue what we do, they have no clue the process of setting up a new test in the lab or the process even just before going out with these tests, it looks like we're underperforming. If someone were to tell me that I was underperforming, it's insulting to me and people who have no clue how anything works in the lab, commenting that we're underperforming by the government. Just pushing, pushing, pushing. But from this government, we're not getting extra pay. We're not getting extra funding. It's just do more with less. - G2A2 (F): It's the nurses and the doctors who get talked about on high regard. You know the people are in the in the labs who are actually pulling the specimens? You need to be able to diagnose conditions or anything? That's never talked to us so that's a stressor for me, personally just knowing how much I do in a shift. For the team I work with how hard every single one of them works. - G1T6 (M): The lack of understanding about lab is incredible. Uh again, I think it's back to that since we're behind the scenes, we're this magical place where specimens get sent and information comes back. I bring it back to people knowing or again, not understanding all, everything that has to go into putting out that quality result at the end of the day.
Subtheme 2: Lack of understanding of their roles, contributing to added demands by leaders	- G2A6 (F): Our supervisor even said they want to increase the number of appointments so that they can get more people in during the day and that just really broke a lot of our spirits 'cause we were all like, “We're giving you everything we have.” - G1T1 (F): We're generally the first place that has to cut their budget. An example is we were told to cut our lab tech training program because it was the longest training program and all of the professions in the hospital and it was actually our CEO that said, “I don't understand it, if you hire a lab tech who's experienced, why do they need 6 weeks of training?” And we had to make them understand that just because you're lab tech at one site, doesn't mean you can go to another and know everything automatically. There's different analyzers at every site. There's different policies and procedures. So many things to learn that it makes the training program quite robust and that's something that senior leadership teams doesn't get because I think for nursing, it's easier. - G2A4 (F): And you're trying to keep still with the stats that the hospital wants you to keep up with uhm 'cause for them, nothing has changed. It's just things are running a lot slower, but numbers are still high. - G1T1 (F): When COVID hit it, pressure was put on us, like “I don't care what you tell me. You need to onboard COVID testing. I don't care if you have no staff, I don't care if you have no money, you're doing it.”

Some participants explained that this lack of understanding extends beyond the government. They noted this lack of understanding from the general public and even other healthcare providers. Participants explained that MLTs could be forgotten because they are non-patient-facing. They also explained that the general public is unaware of patient-facing MLAs like phlebotomists. MLA participants explained that they are often mistaken for a nurse.

Participants expressed that the added demands from management could also result from the limited or unwillingness to understand their roles. They noted that despite the limited resources and staff shortage, expectations from management, whether it be in a hospital or a private clinic, have increased significantly. The pandemic is partly a reason for these high expectations, as MLTs and MLAs are required to complete their regular duties and collect and analyze COVID-19 tests.

### Theme 3: A Poor Work Environment, Exacerbated by the Pandemic

Our results highlighted various factors within the work environment that contributed to the stress of MLTs and MLAs in general and within the context of the pandemic (Table 5). First, there is an emotional component of the job that does not appear to be addressed. Specifically, participants noted that given their role in analyzing specimens, they are the first to know of certain diseases like cancer. While many might not be patient-facing, they indicated that this is a stressful part of their job. For patient-facing MLAs, they reported that they are typically the first line of healthcare staff a patient visits once they receive diagnoses. Unsurprisingly, these workers deal with the patients' distress and poor mental health. Additionally, given the provincial restrictions to curb the spread of COVID-19, patient-facing MLAs noted that they typically work with patients who have been isolated. They listen to their challenges and support them as needed.

**Table 5:** Theme 3: Poor work environment, exacerbated by the pandemic.

Subtheme 1: Unresolved emotions, worsened by the pandemic	- G1T1 (F): We know, peoples' conditions before the physicians know. We know that someone has cancer before it leaves the lab. Being in a small hospital small community, I found myself in situations where I found a family member is going to die. And I had to report those results and maintain my composure and act as if everything is fine. So that aspect itself, which is knowing what we know about the patients can take a toll on a lot of techs. - G2A5 (F): It becomes more than being a phlebotomist. You're almost a therapist to a lot of them working in cancer centers where they're taking blood. You know, we're the first ones they come to once they get a diagnosis and we're the first ones that they cry to once they get a diagnosis, so there's a lot of um emotions there that we deal with as well. - G2A3 (F): Regardless of social distancing, I have held more hands and helped more people in 18 months and wiped the tears of strangers every single day as [other MLAs] have. It's unbelievable because we're the sister that you haven't seen. We're the mother that you can't go see or invite.
Subtheme 2: Increased work demand by taking on COVID-19 testing	- G1T7 (F): The rest of the laboratory is still doing that vital care for the rest of the patients who are actually sick who need routine blood work done and we have to balance them and, and help to keep them positive as well that it's not all about COVID either. We still have sick patients. We still have people who get cancer.
Subtheme 3: Poor communication among healthcare providers and COVID-specific platforms	- G2A2: We do a lot of fact checking for doctors. Yeah. It's just shocking uh, but we do a lot of that because a lot of orders aren't put in correctly. We're missing paperwork. And, We communicate a lot with nurses and there can be a lot of poor communication there in terms of, uhm, what we need to know for us to be able to do our job so that's a stressor for us specific to my role. - G1T3 (F): So we were trying to track down all these COVID-19 results from all these different labs and we're not computer interface with any of these labs. So then it's again… It's a stack of fax reports that come through… - G1T6 (M): The lack of infrastructure and the demands put on us to due to the COVID testing. So here, we were expected to start up these new [COVID] tests. They were throwing instrumentation at us, but everything was coming through on paper requisitions. We're faxing. There's no electronic medical records, so there's the infrastructure for healthcare isn't there to, uhm, facilitate a rapid start of new testing because we're… trying to find couriers to drive around places, where everything is on paper, and being entered manually and results are being back entered into a computer so all of that contributes to stressors and draws on resources that we don't have.
Subtheme 4: Fear of the unknown	- G2A6 (F): I'm very confident. And now with everything going on with COVID, it's kind of uhm, you know, I was very good at this and now I feel like I'm drowning every day. - G2A4 (F): Everybody expressed the employees being overworked, underappreciated, and I find too, that because of that there's a lot of mental health issues that go so unrecognized. - G1T3 (F): The first 4–5 months of the pandemic, I was just existing. If I wasn't at work, my anxiety was terrible, but I could almost put it aside when I was at work and just do what needed to be done. But as like me, the human? It was, it was terrible.

Participants expressed that the workload has increased significantly because of the pandemic. Specifically, in addition to their highly demanding work, MLTs and MLAs were tasked with collecting and analyzing COVID tests, which reportedly has been a stressful experience. This is due to the need to learn new analyzers rapidly and, at the same time, maintain the same pace in terms of collecting and analyzing other specimens that are not related to the pandemic.

Based on the discussions from the focus groups, it appears that the pandemic has contributed to communication issues affecting the work environment. These communication issues exist between medical laboratory employees and other healthcare providers. For instance, MLAs noted that specific tests are sometimes not ordered correctly from the requesting physician or nurse, leading to patient frustration, and added work to the medical laboratory teams.

Communication issues also resulted from not having the right resources (e.g., computer programs) to interact with other necessary departments for COVID-19 testing. Specifically, medical laboratory staff were tasked with completing tasks inefficiently but were expected to complete them quickly. Additionally, they had to learn new instruments for COVID-19 testing, compounding the stress level.

With all the demands in conjunction with the limited resources, participants noted that their mental health has been poorer due to the pandemic. Like other professions, participants explained the “unknown” factor that came with the pandemic along with the restrictions; their mental health deteriorated considerably.

### Theme 4: Resilient and Passionate Group

Despite the considerable challenges, including poorer mental health, MLTs and MLAs presented as passionate and resilient employees (Table 6). Specifically, they offered several coping methods to help them deal with their stressors at work. Exercising was a common coping mechanism among the participants. Similarly, was visiting nature and being outside was also a popular method. Whether from family, friends, colleagues, or management, social support was also noted as a helpful strategy. However, participants emphasized that “alone time” was equally important. They explained that because of their increased demand, need to support others, and the emotional component of their job, being alone was a therapeutic method to recuperate. In addition, seeking mental health support and positive self-talk were also used by some participants.

**Table 6:** Theme 4: A resilient and passionate group.

Subtheme 1: Self-care activities during COVID-19, connecting with others, but appreciating alone time	- G2A3 (F): I'm ripping my mask off at the end of my shift and I'm hiking for 7K, and I don't want to talk to anybody. - G2A1 (F): I've been riding my bike to work. That helps a lot. Riding my bike to work in the morning and riding at home is like really clears things out. But riding my bike with my earphones blasting and just singing, not caring who's watching. With [family], they want to see me, but I have been so stressed that I just want me time. - G2A6 (F): The last thing you want to do sometimes is, even if it's your family or friends, is just have a conversation. And not because there's really nothing else going on, just that question of like, “How is work today?” is like don't get me started. It's the same as it was yesterday and the day before that, and every day 15 months in a row. So, it it's more of a, uh, a trigger. So, definitely having that like alone time… It helps me not think about it, just gives me time to clear my mind.
Subtheme 2: Means to gaining control despite the pandemic	- G2A6 (F): So just trying to find some sort of semblance of control in my own home again, just doing. You know, constantly cleaning out closets and drawers and the fridge. My garden has never been better. I have to really, really focus on that, so that's been uhm, my COVID coping mostly. - G1T3 (F): Taking care of the environment around me. Meaning like that my house is not a mess or. You know there's not a ton of dishes or things like that.
Subtheme 3: Pride and passion for the job	- G1T1 (F): I like to remind myself of why we do it again. It brings us back. We're here for the patients. We're here because we love our job or passionate about it. So, if you remind yourself of that every once in a while, as much as it can't take away all the stress, it does somewhat help put things back in focus. And even though we might not ever see their face, we feel like we know them through their lab work. - G2A2 (F): I personally take a lot of pride in saying I'm a phlebotomist at a hospital. Um, But I do think that with like how much pride I have sit like telling my family or like friends or whoever that I work at a hospital as a phlebotomist. - G1T3 (F): We put the science in medicine and it's very interesting that with such little sample or with such a little part of our patients, we can kind of help the physician and the healthcare team tell the whole story. We're very detail orientated. Our goal is to give out quality meaningful results to our circle of care so that patients can get the right treatment and get on the right path to a healthy life. - G1T6 (M): But even though we're behind the scenes, still, we're not less passionate about patient care. - G2A1 (F): I enjoy it because I enjoy caring for caring for people and for patients and the demographics are quite wide. - G2A3 (F): I don't come with 12 years' experience. I come with 12 years of spit in grit and not leaving until the job is done. I don't leave until the tears are wiped away.

Gaining control was viewed as a COVID-specific coping method. Specifically, participants noted that due to the perceived lack of control from work or because of the imposed restrictions, they strived to gain control in other forms by organizing, cleaning, or focusing on projects like gardening. Some participants indicated that this was a helpful way to improve their mental health and compensate for other areas in their life where control is unmanageable.

Finally, the passion that the MLTs and MLAs have for their profession was viewed as a subtheme related to their resilience. This subtheme emerged organically from their discussions, highlighting that, despite their lack of recognition and the increased demands along with limited resources, they reported a great sense of pride in doing their job, highlighting their high regard for patient-centered care.

## Discussion

This qualitative study revealed general stressors of MLTs and MLAs in Ontario, Canada, and within the context of the pandemic. It also helped us learn about their passion for their work and coping strategies. Our findings revealed substantial stressors evident before the pandemic but have exacerbated since. Due to the notable staff shortage, the behind-the-scenes nature of the job, and challenging working conditions, medical laboratory employees have been experiencing unique and significant stress levels.

Staff shortage is a known yet challenging issue within the healthcare sector (16–18). For instance, a qualitative study revealed that staff shortage was an underlying factor in sickness absenteeism among nurses and healthcare aides (18). As a result of staff shortage, nursing employees often work longer hours with increased demands, which increases their risk of going on sick leave due to physical and mental health reasons. This cyclical paradigm in the healthcare sector is consistent with our study's findings. However, three main factors are making the medical laboratory profession particularly vulnerable.

First, there are few programs and seats available for students interested in enrolling in the medical laboratory field in Ontario. At the same time, there is an increase in employees retiring or leaving the profession due to the stress of the pandemic. Second, there seems to be a missed opportunity for MLAs interested in becoming MLTs. Therefore, opportunities for professional growth in the province are scant, which likely contributes to the disinterest in entering the field at an MLA or MLT capacity. As one participant compared, there are opportunities for career development in the nursing field through bridging programs such as transitioning from a registered practical nurse to a registered nurse. Similar options do not exist in Ontario for the medical laboratory population. A third factor contributing to the staff shortage in the medical laboratory field is the sense of feeling forgotten due to the behind-the-scenes nature of the profession, which is one of the main themes that emerged from this undertaking. As participants noted, MLTs are typically non-patient-facing employees, and MLAs who are patient-facing are mistaken for other positions like nurses. This lack of recognition from the healthcare sector and the government likely contributes to the staff shortage as the public seems vastly unaware of this population. Notably, with limited research in this population, we suspect this lack of recognition is not unique to Ontario but rather a global issue.

In Ontario, MLTs and MLAs were not included in what the government identified as “pandemic pay” (19) to support frontline staff experiencing severe challenges and elevated risk during the COVID-19 pandemic. Their exclusion further exacerbated their frustrations regarding lack of appreciation in the work environment. Like all healthcare workers, MLTs and MLAs are compassionate providers who care about their patients and felt the lack of recognition by the government in acknowledging their contribution as frontline staff providing patient care during the pandemic. The COVID-19 pandemic has significant and widespread influences across all practice areas for medical laboratory professionals. Perturbations in workflow, increases in job demands and occupational stressors have inversely impacted job satisfaction, health, wellbeing, and overall functioning in delivering optimal patient care, thus compromising patient safety.

Pressures from the pandemic exposed the vulnerability of the healthcare system. Before the pandemic, MLTs and MLAs were expected to report to work even if they felt ill, a concept known as presenteeism (20). MLTs and MLAs reported similar issues to healthcare staff like nurses in which they are attending work, feeling concerned about job security (e.g., being sick and still reporting to work), exaggerated levels of attendance that results in illness, occupational stress, work productivity (21), and disability (20). Furthermore, presenteeism has been reported to exacerbate medical conditions (22), damage the quality of working life (23), impact functioning (24), and lead to impressions of ineffectiveness at work due to reduced productivity (25). The relationship between presenteeism and occupational stress may follow a certain dynamic (26). Specifically, pressures placed on relatively scarce health human resources (e.g., recruitment challenges, restricted funding) lead to increased job stress and higher rates of sickness absence. When more MLTs and MLAs are off work, occupational stress increases further, job duties will compound and be viewed as inflexible by workers, and further reductions in service delivery will appear as intolerable by management. The severe consequences for the delivery of health services if MLTs and MLAs are absent lead to elevated thresholds for taking sick leave and result in higher presenteeism levels (26). Strategies are warranted to address sickness absences through evidence-based recommendations that examine their impact on MLTs and MLAs' mental health and work performance.

An added concern for this population is the occurrence of morally injurious events. Morally injurious situations occur when individuals must make decisions that conflict with their values. Specifically, it is the dissonance occurring between what “should” be done and what “could” (or could not) be done (27, 28). This research area remains relatively scant in the extant literature for healthcare workers. However, given the impact of the pandemic on healthcare workers, it has been gaining more traction (29). For medical laboratory employees, facing morally injurious situations occurs primarily due to the guilt of taking time off, recognizing the impact it could have on the remainder of the team, and also the impact it could have on patient care. It also occurs with MLAs working with vulnerable populations who have been unable to seek face-to-face support from their loved ones due to the imposed restrictions to reduce the risk of contagion. To this end, we recommend further research in moral injury in healthcare workers, including occupational groups such as MLTs and MLAs.

Notwithstanding the presented challenges and the lack of recognition, our study revealed that medical laboratory professionals are passionate and patient-centered individuals who presented with positive coping strategies. Despite these positive traits, many are retiring or are leaving the profession, causing a more significant gap in the staff shortage crisis. Thus, moving forward, we recommend that workplaces espouse a culture where MLTs and MLAs feel supported and respected for their contributions to patient care, including the delivery of services. Their roles should be described to the broader healthcare sector, including their work demands, staff shortage, and the emotional aspect of their job to allow for better communication and prioritization of patient needs. While it might be challenging to execute, we recommend more media attention, capturing the work that goes into COVID testing and other specimen analyses to help inform the government and the general public. We also recommend that policymakers and senior managers take a proactive approach. Specifically, they should work collaboratively with MLT and MLA groups to learn about their roles in more detail, allowing for reasonable working demands and realistic expectations.

One main limitation to this study was recruiting enough participants from both groups to form additional focus groups. However, as previously noted, these employees have been facing added pressures, which could have affected recruitment. However, to our surprise, the themes that emerged from both groups were very consistent. Thus, readers should conceptualize these findings at a broader level to understand the challenges faced within the medical laboratory population. Future studies should investigate these groups independently to understand their needs better during the pandemic and beyond.

## Conclusions

MLTs and MLAs are the “conveyer belt” of the healthcare system, as they are involved in the analysis of specimens for diagnostic purposes, yet their contributions are largely unnoticed. While staff shortage and attrition are common issues in the healthcare field, disruptions in medical laboratory services would likely cause significant disruptions to the overall healthcare system. Our study identified key themes that highlight critical knowledge gaps about occupational stressors that are only being raised due partly to the COVID-19 pandemic. Future research studies should explore MLTs and MLAs' lived experiences in their work environment. We believe such an approach will strengthen recruitment and retention capacities for both groups and provide evidence-based information to strengthen government funding to increase MLT enrolment at Ontario Colleges to train the next generation of medical laboratory professionals.

## Data Availability Statement

As this is a qualitative study, releasing the raw data (i.e., transcripts) poses the risk of identifying participants. Thus, due to ethical restrictions, this data is not available. Questions should be directed to: behdin.nowrouzi.kia@utoronto.ca.

## Ethics Statement

The studies involving human participants were reviewed and approved by University of Toronto Research Ethics Board. The patients/participants provided their written informed consent to participate in this study.

## Author Contributions

Both authors substantially contributed to the conception of the work, the interpretation of the data, substantially contributed to the drafting and revising of the work, agreed to be accountable for all aspects of the work in ensuring that questions related to the accuracy and integrity of any part of the work are appropriately investigated and resolved, and approved the final version to be published.

## Funding

 The Medical Laboratory Professionals' Association of Ontario funded the Article Processing Charge of this study.

## Conflict of Interest

The authors declare that the research was conducted in the absence of any commercial or financial relationships that could be construed as a potential conflict of interest.

## Publisher's Note

All claims expressed in this article are solely those of the authors and do not necessarily represent those of their affiliated organizations, or those of the publisher, the editors and the reviewers. Any product that may be evaluated in this article, or claim that may be made by its manufacturer, is not guaranteed or endorsed by the publisher.
