# Supplementary material for: The Forgotten (Invisible) Healthcare Heroes: Experiences of Canadian Medical Laboratory Employees Working During the Pandemic
Source: Front Psychiatry. 2022 Mar 16;13:854507. doi: 10.3389/fpsyt.2022.854507 (PMC8966580; doi:10.3389/fpsyt.2022.854507)
Supplement: Supplementary file 1 [file Data_Sheet_1.docx]

Supplementary Material

# Appendix A: Interview Guide

Authors: Dr. Basem Gohar & Dr. Behdin Nowrouzi-Kia

***Before the focus group with the participants:***

The session commences by a short meet and greet to build rapport. Participants are reminded of the study’s purpose, risks, and benefits, which were detailed in their consent form. The moderator reminds participants that the session will be audio recorded for accuracy of data collection.

**Questions:**

1. Tell us about your job duties as a medical laboratory professional.
2. In general, what are the common stressors of your job?
   1. Follow-up questions as necessary
3. Tell us what are the stressors that manifested during the pandemic
   1. Follow-up questions as necessary
4. Tell us how you found ways to manage stresses you’ve experienced while working as a medical laboratory professional
   1. Follow-up questions as necessary
5. Is there anything else you would like to add?

***After the focus group with the participants:***

Checking in with participants and receiving feedback.
