# Supplementary material for: The Forgotten (Invisible) Healthcare Heroes: Experiences of Canadian Medical Laboratory Employees Working During the Pandemic
Source: Front Psychiatry. 2022 Mar 16;13:854507. doi: 10.3389/fpsyt.2022.854507 (PMC8966580; doi:10.3389/fpsyt.2022.854507)
Supplement: Supplementary file 2 [file Data_Sheet_2.pdf]

Supplementary Material

Appendix B: Quality Assessment

Authors: Dr. Basem Gohar & Dr. Behdin Nowrouzi-Kia

Checklist: Consolidated criteria for reporting qualitative research (COREQ): a 32-item checklist for interviews and focus groups (Tong et al., 2007)

1. Basem Gohar
2. Basem Gohar = PhD, MSc, C. Psych; Behdin Nowrouzi-Kia = PhD, OT Reg
3. Basem Gohar = Assistant Professor & Clinical Psychologist; Behdin Nowrouzi-Kia = Assistant Professor & Occupational Therapist
4. Basem Gohar = Male/Man; Behdin Nowrouzi-Kia = Male/Man
5. Both researchers have extensive research in working with healthcare providers and qualitative research
6. Yes. Researchers spoke with the participants prior to the study. Also, at the beginning, each person had a chance to introduce themselves within the group to foster a warm and welcoming focus group.
7. Participants were fully aware of the researchers' research interests and their clinical experience. Participants were also aware of the study's focus.
8. The interviewers recognize that through their clinical and research work that mental health was likely impeded by the pandemic. However, using an inductive approach as well as using open-ended questions, it allowed for the participants to describe their experiences free of bias from the researchers.
9. The study was a qualitative descriptive study
10. We recruited at the provincial level. Interested participants contacted the researchers directly.
11. Participants were approached by email (response to their email)
12. A total of 13 who were involved in the study (Seven medical laboratory technologists and six medical laboratory technicians/assistants).
13. For the medical laboratory technologist group there were a total of 10 interested participants. Of those, seven participated. Of the three who did not attend, two did not respond to follow-up emails, and one could not attend due to a conflict in schedule. For the medical laboratory technicians, assistants, there were also 10 who were interested. Six of those participated. There were two who did not respond to follow-up emails, one could not attend due to a conflict in schedule, and one who was unable to participate due to connectivity issues.
14. Data were collected securely VIA Microsoft Teams
15. No
16. Gender, geographic location, and work setting were collected. Of the 13 participants, 11 identified as women and two as men. One participant was from Peterborough area, two were from Hamilton Region, one was from Cochrane District, one was from Ottawa region, two were from Simcoe region, one was from Middlesex/Thames region, one was from Durham region, one from Niagara region, and three were from the Greater Toronto Area. Of those, four were identified working in rural regions. Seven work exclusively in a hospital setting while six work in a clinic

or a private laboratory setting. Three identified they work in multiple settings. Work experience varied from 1 year to 32 years.

17. The questions were created and prompted by the authors. It was not trialed on this group. This is because we adapted an interviewing guide from another study with a different healthcare population that was well received.
18. Focus groups were not repeated.
19. Data were audio recorded.
20. Yes, but the focus remained with our audio data which was transcribed verbatim.
21. Each focus group took approximately 75 minutes.
22. Due to low enrolment, we were unable to do further recruitment. However, there was strong consistencies between the two groups and thus we feel that we reasonably reached saturation.
23. Transcripts were not returned due to the nature of focus groups. However, one method to ensure accuracy was the moderator (researcher) paraphrased each response, allowing the participant to ensure accuracy during the focus group.
24. Two.
25. Yes, see tables.
26. Themes were inductive as such were derived from the data.
27. We used the Software Quirkos for managing, coding, and analyzing data.
28. No.
29. Yes, we presented quotations. Yes, participants numbers were identified.
30. Yes, data was very consistent.
31. Yes.
32. Yes.
